# The Plea of Insanity

**Published:** 1898-03

**Authors:** 


### THE PLEA OF INSANITY.

The jailer of our county jail in Atlanta has recently made grievances to the effect that the jail is becoming more of a lunatic asylum than a repository for straight-out criminals. This is a lamentable fact, yet nevertheless true. Criminals are criminals before the bar of justice, but ere their trial is over they are raving lunatics. Surely this age of ours is the age for the production of insane persons. Certainly would we conjecture that such is the case should we follow the results obtained in our criminal courts. It is indeed a sad condition of affairs that atrocious criminals can cover their heinous deeds under the cloak of insanity and then seek the repose and comforts of a well-conducted asylum! We have no sympathy with the justice which can recognize a deed of murder and then palliate it as an act of an insane moment. Who would not become insane had he shot a man down in cold blood or killed a defenseless woman? The plea of insanity on the part of the lawyers for a criminal is becoming too threadbare. If such a condition of affairs continues, justice will never have a victim. It is said that the term *neurasthenia* is entirely of American coinage, since it is supposed to represent a condition of the nervous system common only to Americans. It will soon be said that *homicidal insanity* is purely an American production, and will be placed in our dictionaries as "an American term representing that condition of the nervous system at the time one man kills another." The medical profession must stop this lamentable state of affairs by giving conscientious testimony on the witness-stand. We believe that the plea of insanity should not be allowed to exist unless unimpeachable testimony shows that the culprit was insane before the murderous deed was committed. The very fact that the man or woman becomes insane when the act is committed, or manifests insanity after the deed is done, is self-evident that he has enough conscience to recognize the enormity of the crime, or knowledge enough to know that he is guilty of murder in the eyes of the law. Besides, how do we know but that the asylum in which such criminals are placed will yet be the scene of more murder enactments? A case of this kind has but recently occurred in our own State asylum at Milledgeville. B. H. Osborne, a murderer, was adjudged insane (?) after shooting down a man in cold blood, and as a result of the verdict he was placed in the State asylum. A few weeks ago the "less insane" of the inmates, among which number was Osborne, were permitted more freedom in their Christmas enjoyment, and were having an entertainment, when Osborne suddenly bolted by the guards, shooting at them as he went, and in a few moments gained his liberty. The superintendent, Dr. Powell, being accidentally near by Osborne in his flight, narrowly escaped a flying bullet aimed at him. Osborne had sufficient knowledge to make good his escape. Now, as we well know, the asylum at Milledgeville is one of the best-managed in this country, and what happened there would be liable to happen at any similar institution. In other words, there comes a time in the confinement of individuals in asylums when they are thought to have become harmless, and in these moments they are given more liberty. This management is rational, but often serious in its results. Such class of the "insane criminals" should be kept in their own prison cells, if the justice of the law has decreed that our asylum shall be the repository for such beasts. Let justice be swifter in its administration. The great agitation about the "lynch law" is just as applicable here. Criminals appeal from the lower to the higher courts, and by the time the last one is reached the prisoner is "absolutely insane." If there is doubt enough about the matter to stay justice in a human execution, then make it a life-sentence and let the State and the country make the criminal a producer and not an expense on its treasury. Above all, let our "experts" give testimony according to facts and not according to the side they are on, and then will justice be properly meted to criminals and this farce play of insanity be stripped of its mask. R.

